# Dietary nitrate and resveratrol co‐supplementation prevent HFD‐mediated impairments in carotid blood flow and behavioral parameters in male mice

**DOI:** 10.14814/phy2.70814

**Published:** 2026-03-24

**Authors:** Geneviève J. DesOrmeaux, Jessica L. Dowling, Pierre‐Andre Barbeau, Rachel M. Handy, Melanie Alpaugh, Graham P. Holloway

**Affiliations:** ^1^ Department of Human Health Sciences University of Guelph Guelph Ontario Canada; ^2^ Department of Molecular and Cellular Biology University of Guelph Guelph Ontario Canada

**Keywords:** brain blood flow, cortex, mitochondrial bioenergetics, nutrition

## Abstract

While dietary nitrate (NIT) and resveratrol (RSV) have known metabolic effects within peripheral tissues, their impact within the brain remains unknown. We investigated these compounds' independent and combined (+RN) efficacy in preserving brain blood flow, and their impact on mitochondrial bioenergetics and behavior in C57Bl/6N male mice consuming a high‐fat diet (HFD; *n* = 100). Carotid blood flow was measured via ultrasonography, the cortex was permeabilized to determine mitochondrial function, and homogenized for analysis of various protein targets. Physical activity in a novel environment was evaluated as an index of habituation outcomes. HFD feeding reduced carotid blood flow and decreased cortical VEGF protein content (*p* = 0.057) but did not alter mitochondrial function. While RSV did not prevent HFD‐induced declines in brain blood flow, NIT independently, and combined with resveratrol (+RN), preserved carotid blood flow (*p* < 0.02) and VEGF protein content. These indexes of brain blood flow coincided and correlated with reduced anxiety‐like behavior. Despite the improved habituation, combined +RN supplementation reduced cortical mitochondrial respiration without altering mitochondrial ROS emission, redox balance, or markers of mitochondrial content/mitophagy. These findings suggest NIT may have beneficial effects within the brain through the maintenance of carotid blood flow, as opposed to alterations in mitochondrial bioenergetics within the cortex.

## 1 INTRODUCTION

The brain is a highly metabolically active organ that receives a significant proportion of oxygen delivery. Despite only representing ~2% of body weight, the brain receives ~20% of total blood flow and accounts for ~20% of resting energy expenditure, underscoring the organ's high metabolic demand (Clarke & Sokoloff, 2023). This substantial blood supply is critical given the brain's limited capacity for energy storage and reliance on continuous oxygen and nutrient delivery for proper function (Norat et al., 2020; Roy & Sherrington, 1890; Smith et al., 2002). Cerebral blood flow is a dynamically regulated process which can promote targeted regional perfusion within neuronally active regions (Attwell et al., 2010; Petzold & Murthy, 2011). This neurovascular coupling can be partially attributed to the release of vasoactive substances, including nitric oxide (NO) (Attwell et al., 2010). When this regulation is compromised, reductions in perfusion initiate a cascade of cellular signaling whereby prolonged ischemic conditions have been implicated in various neurodegenerative conditions including Alzheimer's disease and other forms of dementia, with these pathologies showing a clear relationship between reduced blood flow and cognitive impairments (Ceylan, 2023; Graff et al., 2023; Xing et al., 2012).

At the cellular level, this robust blood flow serves as the primary delivery mechanism to mitochondria, which are essential for aerobic energy production, neurotransmission, dendritic growth/neurogenesis, and maintenance of overall neuronal health within the brain (Calabrese et al., 2001; Rangaraju et al., 2019). Altered mitochondrial bioenergetics, characterized by a reduction in oxidative phosphorylation (OXPHOS) and increased production of reactive oxygen species (ROS)/redox stress, has emerged as a pathogenic mechanism in various neurological conditions (Angelova & Abramov, 2018; Chan, 2006). The brain's high metabolic demand makes it particularly sensitive to dietary patterns that alter substrate availability and vascular function. High fat diet (HFD) consumption is linked to cognitive impairments through multiple mechanisms including insulin resistance and lower arterial blood flow, compromised blood–brain barrier (BBB) permeability, and changes in peripheral mitochondrial function (Cavaliere et al., 2019; Karmi et al., 2010). While studies have documented HFD‐induced post‐translational modification of OXPHOS‐related proteins (Siino et al., 2021), the direct effect on mitochondrial respiration in permeabilized brain tissue–a methodology which preserves a more native structure compared to isolated mitochondria–remains incompletely characterized. This gap highlights the need for both more precise characterization of HFD's effects on brain bioenergetics and exploration of potential therapeutic approaches to mitigate these effects.

Resveratrol (RSV) and nitrate (NIT) represent two promising nutritional compounds that influence cellular metabolism through distinct but potentially complementary mechanisms. RSV, a polyphenol found in various plant sources such as the skin of red grapes, berries, and peanuts (Lyons et al., 2003; Sobolev & Cole, 1999; Wang et al., 2002) has the capacity to cross the BBB due to its lipophilic properties. RSV interacts with several signaling pathways that regulate gene transcription and mitochondrial biogenesis, including peroxisome proliferator‐activated receptor gamma coactivator 1‐alpha (PGC1α), 5′‐adenosine monophosphate‐activated protein kinase (AMPK), and Sirtuin 1 (SIRT1) (Lin et al., 2010; Price et al., 2012; Rege et al., 2014; Vauzour, 2012). Similarly, dietary NIT, abundant in green leafy vegetables and beetroot, undergoes serial reduction to produce NO, affecting cellular metabolism (Petrick et al., 2023; Dezfulian et al., 2016; Shiva et al., 2007). While NIT's effects on mitochondrial bioenergetics have been characterized in peripheral tissues (Brunetta et al., 2020; Brunetta et al., 2022; Cordero‐Herrera et al., 2019; Govoni et al., 2008; Peleli et al., 2020), and require SIRT1 (Brunetta et al., 2022), its impact on brain cellular processes requires further investigation. However, dietary nitrate has been shown to enhance cerebral blood flow, particularly in the frontal cortex (Presley et al., 2011; Wightman et al., 2015) and improve arterial stiffness (Pinheiro et al., 2025). Indeed, these compounds have been associated with increased vascular endothelial growth factor (VEGF) expression and upregulation of endothelial nitric oxide synthase (eNOS), suggesting overlapping mechanisms that influence vascular function and angiogenic processes (Simão et al., 2012; Zhang et al., 2003). Given the potential for these bioactive nutritional compounds to influence neurological function and behavior through complementary mechanisms affecting both nutrient delivery (blood flow) and cellular energy metabolism, we aimed to: (i) solidify the effects of a HFD on brain oxidative metabolism and blood flow and (ii) determine the efficacy of independent and combined supplementation with dietary NIT and RSV to modulate oxidative phosphorylation and behavioral parameters within HFD‐fed mice. We hypothesized that alterations in blood flow to the brain, mitochondrial respiration, and mitochondrial ROS induced by HFD would be modulated with combined RSV + NIT supplementation, leading to changes in behavior. This approach allows us to examine both the individual and potential synergistic effects of these nutritional compounds on brain metabolism and function in the context of diet‐induced metabolic stress.

## 2 METHODS

### 2.1 Experimental design

100 C57Bl/6N male mice were purchased from Charles River Laboratories (Senneville, QC, Canada) at six weeks of age and separated into two separate experimental cohorts. Only male mice were utilized in this study; however, this limits the interpretation of the findings to male mice. All mice were housed at the University of Guelph (22°C) on a 12 h light: dark cycle with 24 h access to food and water. Within the first experimental study, 20 mice aged 10 weeks were randomized into two groups fed with a low‐fat diet (LFD; 10% energy from fat, Research Diets, cat. no. D12450) or a high fat diet (HFD; 60% energy from fat, Research Diets, cat. D12492) for 8 weeks. 1 HFD‐fed mouse died during the last week of the dietary intervention and was removed from further analyses. Within the second experimental study 80 mice aged 10–14 weeks were randomized into one of four HFD‐fed groups for 8 weeks. Unsupplemented (HFD), supplemented with +RSV (172 mg RSV/kg diet; Sigma R5010), supplemented with +NIT (4 mM sodium nitrate via drinking water; Sigma S5506), or supplemented with both RSV and NIT (+RN). The dose of RSV and NIT was determined from previously published literature which demonstrated a positive metabolic effect in obese models (Brunetta et al., 2022; DesOrmeaux et al., 2021; Handy et al., 2023; Monaco et al., 2018; Price et al., 2012; Smith et al., 2013). Animals were group housed (*n* = 3/cage) and based on average food and water intake within a cage equates to approximate ingestion of ~10 mg·kg body wt^−1^·day^−1^ of RSV and ~20 mg·kg body wt^−1^·day^−1^ of NIT, independent of individual or combined supplementation as previously reported (Handy et al., 2023). Indices of peripheral tissue metabolism (adipose tissue, liver and muscle) have previously been reported in a subset of mice from the present study (Handy et al., 2023). All diets were purchased from Research Diet (New Brunswick, NJ, USA).

### 2.2 Animal ethics statement

All experiments were performed in accordance with the University of Guelph Animal Care Committee (AUP No. 4241).

### 2.3 Glucose tolerance test

To confirm the whole‐body metabolic consequences of an HFD, an intraperitoneal glucose tolerance test (ipGTT; 2 g/kg body weight) was conducted after a 4 h fast (8:00–12:00 h) in LFD vs. HFD animals over the final week of dietary intervention. Blood glucose levels were determined from the tail vein at 0, 15, 30, 45, 60, 90, and 120 min post injection using commercially available glucose strips (FreeStyle Lite NDC 99073‐0708‐27, Abbott Diabetes Care, Alameda, CA). The area under the curve (AUC) was calculated as the total incremental area under the curve versus time as previously reported (Snook et al., 2016). We have not reported ipGTT results from the supplemented HFD‐fed cohort (HFD, +RSV, +NIT, and +RN), as glucose tolerance data from these animals have been published previously (Handy et al., 2023).

### 2.4 Carotid ultrasonography

Carotid ultrasonography measurements were carried out as previously described (Kenwright et al., 2015). Briefly, the animals were anesthetized with isoflurane‐oxygen (4%, 1 L/min) in a sealed chamber and were transferred to a heated platform in supine position where anesthesia (1.5%–2%, 1 L/min) was maintained below the level of pedal reflex. Body temperature was maintained at 37.0°C (± 0.5°C) during the entire examination and heart rate was continuously monitored to ensure that the observed blood flow differences were not attributable to variations in heart rate. Ultrasound scanning was performed using the Vevo770 imaging system (VisualSonic, Toronto, ON, Canada) and analyzed using the tools provided in the manufacturer's software (Vevo2100 Workstation software package). High‐resolution images were taken across the left parasternal long axis using the MS550D ultrasound transducer (at a frequency of 32 MHz). Specifically, B‐mode was used to position the transducer 1‐2 mm from the carotid bifurcation, ensuring consistent measurement location across animals to minimize variability. Carotid artery cross‐sectional area was calculated from B‐mode measurements using the formula A = π*r* ^2^. Pulse wave Doppler echocardiography was then performed with gain settings of 32–35 dB to determine the velocity‐time integral (VTI). From the spectral Doppler waveforms, peak systolic velocity was measured. The pulsatility index was calculated as (PSV‐EDV)/mean velocity to assess vascular resistance. Carotid flow was calculated as the product of area and VTI multiplied by heart rate. A beam angle <60° was used, along with a corresponding correction factor to account for estimation errors in VTI due to geometric spectral broadening of the carotid artery, as described previously (Kenwright et al., 2015).

### 2.5 Tissue collection and euthanasia

After 8 weeks of treatment, mice were anesthetized using sodium pentobarbital (60 mg/kg bodyweight). The cortex was obtained, followed by cardiac excision for concurrent (unpublished) study, thereby ensuring euthanasia. The cortex was immediately placed in ice‐cold BIOPS preservation buffer (50 mM MES, 7.23 mM K_2_EGTA, 2.77 mM CaK_2_EGTA, 20 mM imidazole, 0.5 mM dithiothreitol, 20 mM taurine, 5.77 mM ATP, 15 mM PCr, and 6.56 mM MgCl_2_·H_2_O, pH 7.1) for mitochondrial respiration and H_2_O_2_ measurements, while the other portion of the cortex was immediately flash frozen in liquid nitrogen for western blotting and citrate synthase (CS) activity measurements.

### 2.6 High‐resolution respirometry

Mitochondrial respiration was measured in permeabilized tissue as previously reported (Herbst & Holloway, 2015). The use of permeabilized intact tissue was chosen for this study as opposed to brain homogenates and/or isolated mitochondria as it provides a heterogenous mitochondrial population and prevents mechanical disruptions/preserves the structural integrity/native mitochondrial properties (Dubinsky, 2009; Herbst & Holloway, 2015; Picard et al., 2011). Briefly, the cortex in BIOPS was further sectioned in 2–3 mg pieces under a microscope, weighed and placed in ice‐cold MiR05 (0.5 mM EGTA, 3 mM MgCl_2_·6H_2_O, 60 mM K‐lactobionate, 10 mM KH_2_PO_4_, 20 mM HEPES, 110 mM sucrose, 1 g/L free fatty acid (FFA) BSA, pH 7.1). Samples were then placed in high‐resolution respirometer (Oroboros Oxygraph‐2K; Oroboros instruments, Innsbruck, Austria) containing MiR05 at 37°C, with saturating room air and constantly stirring at 750 rpm. Samples were provided 10 min to equilibrate prior to the addition of 50 μM saponin (Sigma 47036) for permeabilization. Rate of oxygen flux was then measured following the addition of 5 mM pyruvate (Sigma P2256), 2.5 mM malate (Sigma M1000), 25 μM ADP and 5 mM ADP (Sigma A2754), 10 mM glutamate (Sigma G1626), and 10 mM succinate (Sigma S2378) in succession and normalized to wet weight (ww) and CS activity.

### 2.7 Citrate synthase activity

CS activity was determined spectrophotometrically similarly to previously reported (Holloway et al., 2007). Briefly, cortical tissue samples (6–10 mg) were homogenized in a solution (0.1 M KH_2_PO_4_ and 0.05% BSA, pH 7.3) at a ratio of 100 μL solution per mg of tissue. Enzyme activity was measured spectrophotometrically at 37°C at 412 nm. CS activity was calculated using an extinction coefficient of 13.6 mM^−1^ cm^−1^ and expressed as IU/g tissue weight (IU = μmol/min).

### 2.8 Reactive oxygen species emission

Mitochondrial H_2_O_2_ was assessed fluorometrically, similar to previous reports (Brunetta et al., 2020; DesOrmeaux et al., 2021). The cortex was sectioned in 2 mg pieces under a microscope, weighed, and placed in ice‐cold Buffer Z (105 mM K‐MES, 30 mM KCl, 1 mM EGTA, 10 mM KH_2_PO_4_, 5 mM MgCl_2_, 5 μM glutamate, 5 μM malate, and 0.5% FFA‐free BSA (BioShop ALB006), pH 7.1) containing 10 μg/mL digitonin (Sigma D5628), 1 U/mL horseradish peroxidase (HRP; Sigma P8375), 40 U/mL superoxide dismutase (SOD; Sigma S8160), and 10 μM Amplex Red (Invitrogen A36006). Mitochondrial H_2_O_2_ emission rates were determined in the presence of 20 mM succinate (Sigma S2378) and 100 μM ADP (Sigma A2754) added in succession. This protocol was repeated for a representative trace in the presence and absence of 166.7 μM 2,4‐dinitrophenyl hydrazine (DNP; Sigma 34,334), a mitochondrial uncoupler, to confirm mitochondria as the source of H_2_O_2_ (Paglialunga et al., 2015).

### 2.9 Western blotting

The cortex was homogenized in lysis buffer, diluted to 1 μg/μL, and loaded equally onto standard SDS‐PAGE gels as previously reported (Herbst et al., 2018; Herbst & Holloway, 2015). Primary antibody targets include: phosphorylated 5′‐adenosine monophosphate‐activated protein kinase (pAMPK_Thr172_; 1:1000, Cell Signaling Technology Cat# 2535), total AMPK (tAMPK; 1:1000, Cell Signaling Technology Cat# 2757), adenine nucleotide translocase 1 (ANT1; 1:1000, Abcam Cat# ab110322), phosphorylated eukaryotic elongation factor 2 (p‐eEF2; 1:1000, Cell Signaling Technology Cat# 2331), total‐eEF2 (1:1000, Cell Signaling Technology Cat# 2332), endothelial nitric oxide synthase (eNOS; 1:1000, Abcam Cat# ab5589), trans‐4‐hydroxy‐2‐nonenal (4HNE; 1:1000, Alpha Diagnostics Cat# HNE‐11s), microtubule‐associated protein 1A/B‐light chain 3 I/II (LC3‐I/II; 1:100, Cell Signaling Technology Cat# 4108), phosphorylated mammalian target of rapamycin (p‐mTOR; 1:1000, Cell Signaling Technology Cat# 2971), total‐mTOR (1:1000, Cell Signaling Technology Cat# 2972), nitrotyrosine (3‐NT; 1:500, Cayman Chemical Cat# 189542), OXPHOS cocktail (1:500, Abcam Cat# ab110413), Parkin (1:1000, Cell Signaling Technology Cat# 4211), PGC1α (1:1000, Calbiochem Cat # 516557), PTEN induced putative kinase 1 (PINK1; 1:1000, Cell Signaling Technology Cat# 6946), sirtuin 1 (SIRT1; 1:1000, Upstate Cat# 07‐131), and vascular endothelial growth factor (VEGF; 1:1000, Abcam Cat# ab46154). All membranes were imaged using enhanced chemiluminescence (ChemiGenius2 Bioimaging System #1705061, SynGene, Cambridge, UK), quantified with the FluorChem HD imaging chemiluminescence (Alpha Innotech). Ponceau staining was used as a loading control.

### 2.10 Behavioral index analysis

To evaluate physical activity as an index of cognition and anxiety, mice were placed within the Comprehensive Lab Animal Monitoring System (CLAMS; Columbus Instrument, Columbus, OH, USA) for 24 h. During this entire period, the physical activity of mice was measured using light beam breaks quantifying total horizontal movement. For anxiety assessment, activity was analyzed during the first 40 min of exposure to the novel environment, with the activity change ratio calculated as (total activity end)/(total activity end + total activity start) during this period. Lower initial activity indicates higher anxiety‐like behavior (Bolivar, 2009; Heinz et al., 2021; Masnata et al., 2019). Habituation was similarly evaluated through the activity change ratio using the same formula but applied to the 60–100 min period, where a greater decrease in exploratory behavior indicates enhanced habituation through growing environmental familiarity (Bolivar, 2009; Heinz et al., 2021).

### 2.11 Statistical analysis

Statistical analyses were performed using GraphPad Prism 10 (GraphPad Software, La Jolla, CA). All values are reported as mean ± SD unless otherwise stated. Data points that deviated more than two standard deviations from the mean were considered outliers and excluded from analysis. Data were assessed for normality using the Shapiro–Wilk test. For data that passed the Shapiro–Wilk test, LFD versus HFD comparisons were analyzed using a two‐tailed Student's *t*‐test, while the HFD, +RSV, +NIT, +RN comparisons were analyzed using one‐way ANOVA followed by Tukey post hoc test where appropriate. Time course data that passed normality were analyzed using two‐way ANOVA followed by Tukey post hoc test where appropriate. For data that did not pass the Shapiro–Wilk test, LFD versus HFD comparisons were analyzed using Mann–Whitney test, while HFD, +RSV, +NIT, +RN comparisons were analyzed using Kruskal–Wallis test followed by Dunn's multiple comparison test. Simple linear regression analysis was used to determine the association (*r*) between habituation indexes and carotid blood flow. Significance was assumed when *p* < 0.05.

## 3 RESULTS

### 3.1 The effects of HFD feeding on whole‐body parameters and brain hemodynamics

**FIGURE 1:**
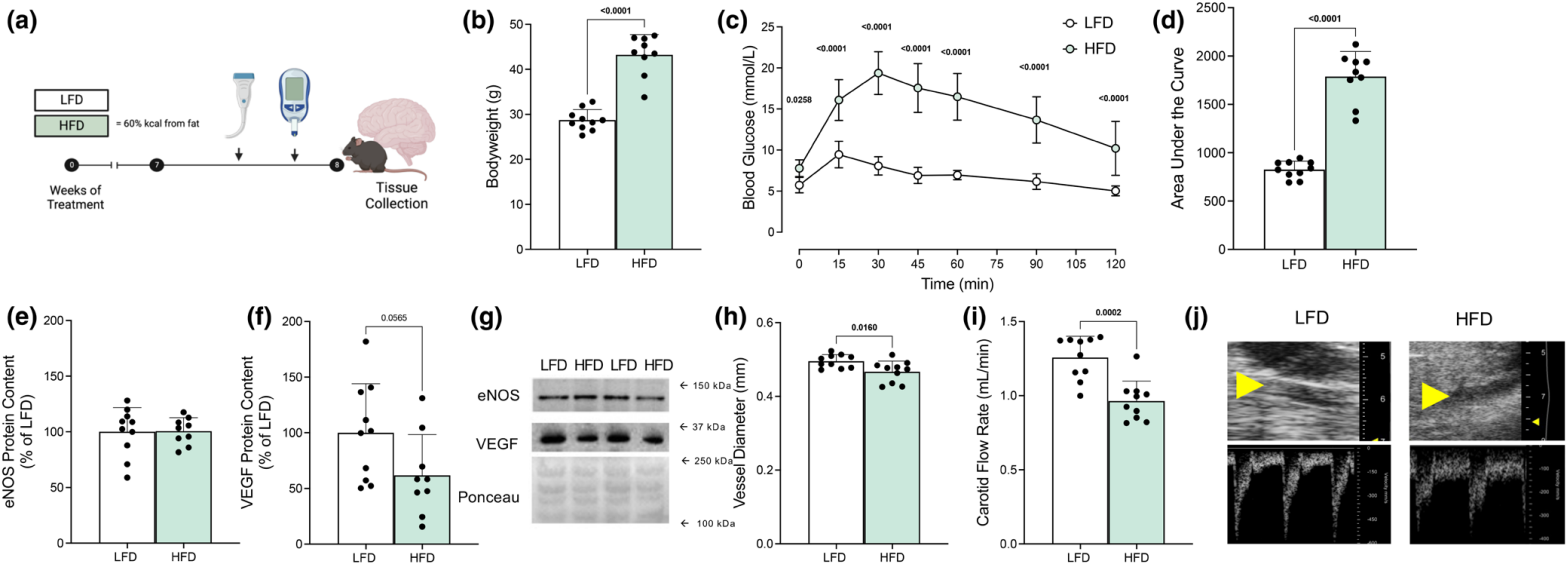
HFD feeding promotes whole body glucose intolerance and impairs blood flow to the brain (*n* = 9–10 mice/group). Experimental approach in male mice (C57Bl6/N) aged 10 weeks at the onset of treatment (a, created in BioRender). Bodyweight at time of euthanasia (b). Glucose tolerance test (GTT) blood glucose level at each time point (c), and calculated area under the curve (d). Protein content of eNOS (e) and VEGF (f) quantified as a percentage of LFD control group from the cortex region of the brain and representative western blots (g). Carotid artery diameter (h), and carotid flow rate (i) obtained by carotid ultrasonography with B‐mode (yellow arrow indicate carotid artery) and doppler ultrasonographic representative images (j). Data expressed as mean ± SD. All data met the assumption of normality as assessed by the Shapiro–Wilk test. All data were analyzed using a student's two‐tailed *t*‐test except the GTT time‐course measurements which were compared by two‐way ANOVA with Tukey post hoc test where appropriate. Bolded *p* values are indicated for comparisons that reached statistical significance (*p* < 0.05). eNOS, endothelial nitric oxide synthase; HFD, high fat diet; LFD, low fat diet; VEGF, vascular endothelial growth factor.

We first aimed to establish the basic effects of consuming an HFD on coordinated oxidative phosphorylation in the brain. As expected, HFD‐feeding caused significant weight gain (Figure 1b, *p* < 0.0001) and the development of glucose intolerance (Figure 1, c and d, *p* < 0.0001), indicating the induction of a diet‐induced obesity phenotype. To assess potential vascular changes following the HFD‐feeding period, we examined markers of angiogenic signaling and utilized carotid ultrasonography to determine unilateral carotid blood flow. While cortex eNOS protein content was unchanged (Figure 1e, *p* > 0.10), HFD‐feeding tended to reduce VEGF content within the cortex (Figure 1f, *p* = 0.057) and significantly reduced left carotid vessel diameter and carotid flow rates (Figure 1h–j, *p* < 0.02). These reductions in diameter and flow rates indicate the changes in vascular function via this vessel, while the trend for reduced VEGF may reflect altered angiogenic potential; however, we observed no changes in downstream protein, eNOS. These findings demonstrate that HFD consumption alters carotid vascular flow, though the relationship between carotid flow and overall cerebral perfusion requires further investigation.

### 3.2 The effects of HFD‐feeding on mitochondrial bioenergetics and markers of metabolic homeostasis

**FIGURE 2:**
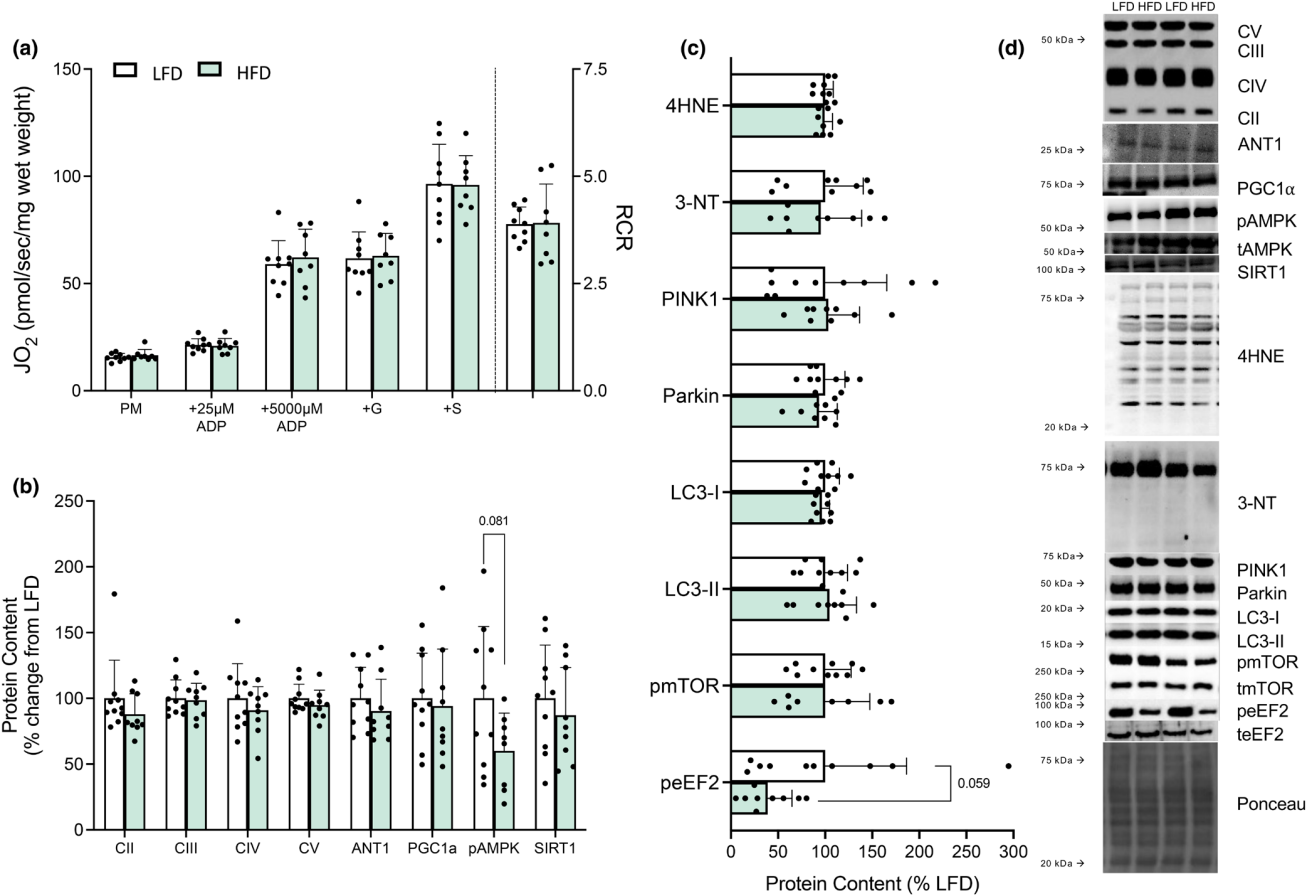
HFD feeding does not alter mitochondrial content and respiration, nor markers of oxidative stress and protein homeostasis in the cortex of the brain (*n* = 8–10 mice/group). Mitochondrial respiration in the presence of various submaximal ADP and saturating complex I‐ and II‐linked substrates (a). Protein content of OXPHOS complexes (CII, CII, CIV, CV), ANT1, PGC1α, the ratio of phosphorylated: total AMPK, and SIRT1 (b) quantified as a percentage of LFD control group. Protein content of 4HNE‐modified protein, 3‐NT, PINK1, parkin, LC3‐1, LC3‐II, as well as the ratio of phosphorylated: total mTOR and eEF2 (c) quantified as a percentage of LFD control group in the cortex of the brain and representative western blots (d). Data expressed as mean ± SD. Data expressed as mean ± SD. Data were assessed for normality using the Shapiro–Wilk test. All data which met the assumption of normality were analyzed using a student's two‐tailed *t*‐test while the remaining data that did not pass the normality test were analyzed using Mann–Whitney test (A–PM; B–CII, CV, ANT1). Bolded *p* values are indicated for comparisons that reached statistical significance (*p* < 0.05). ADP, adenosine diphosphate; AMPK, 5′‐adenosine monophosphate‐activated protein kinase; ANT1, adenine nucleotide translocase 1, eEF2, eukaryotic translation, elongation factor 2; +G, +glutamate; HFD, high fat diet; JO_2_, rate of oxygen consumption; LFD, low fat diet; LC3, microtubule‐associated protein 1A/1B light chain 3; mTOR, the mammalian target of rapamycin; 3‐NT, nitrotyrosine; OXPHOS, oxidative phosphorylation; PINK1, PTEN‐induced kinase 1; PGC1α, peroxisome proliferator‐activated receptor gamma coactivator 1‐alpha; PM, pyruvate + Malate; respiratory control ration; +S, +succinate; SIRT1, sirtulin 1; 4HNE, 4‐hydroxynonenal.

Given the HFD‐mediated reduction in carotid blood flow, we assessed various parameters of mitochondrial bioenergetics and redox balance within the cortex. HFD‐feeding did not alter leak respiration (PM), submaximal (+25 μM ADP) or maximal (+5 mM ADP) respiration, maximal OXPHOS (complex I + II) or the respiratory control ratio (RCR) (Figure 2a, *p* > 0.10). In support of these data, there were no differences in mitochondrial protein content (CII, CIII, CIV, CV; Figure 2b, *p* > 0.10), PGC1α, or SIRT1, though the ratio of pAMPK: tAMPK showed a trend towards a reduction with HFD feeding (Figure 2b, *p* = 0.081). Analysis of redox balance, including levels of 4HNE modified proteins (a marker of lipid peroxidation; Figure 2c, *p* > 0.10) and 3‐NT protein content (readout of NO‐dependent protein modification; Figure 2c, *p* > 0.10) revealed no differences between groups. Additionally, the content of PINK1, Parkin, LC‐I, and LC‐II, representing key mitophagy proteins, and the ratio of phosphorylated: total mTOR (marker of protein synthesis), were not different following HFD‐feeding (Figure 2c, *p* > 0.10). In contrast, the ratio of phosphorylated: total eEF2 displayed a strong trend for a reduction following HFD‐feeding (Figure 2c, *p* = 0.059) suggesting a possible attenuation in protein synthesis. Taken together, these results indicate that despite a reduction in carotid blood flow, mitochondrial respiration and oxidative stress remain unaltered in the cortex of HFD‐fed mice.

### 3.3 The effects of +RSV and +NIT supplementation on attenuating HFD‐mediated reductions in carotid blood flow and markers of angiogenesis in the cortex

**FIGURE 3:**
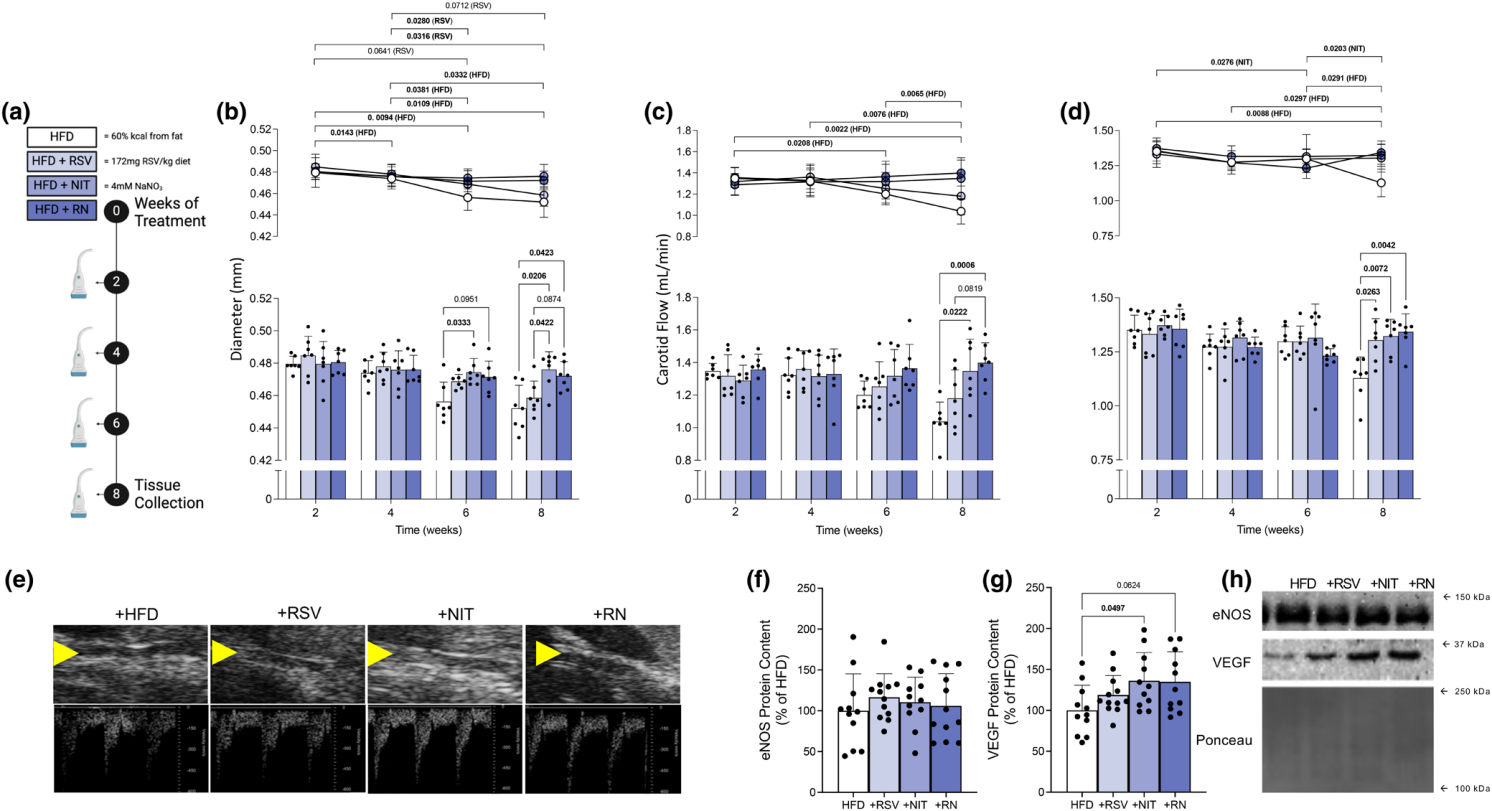
Eight weeks of supplementation with +NIT and +RN prevents the HFD‐mediated decrease in blood flow to the brain (*n* = 7–12 animals/group). Experimental approach in male mice (C57Bl6/N) aged 10 weeks at the onset of treatment (a, created in BioRender). Carotid artery diameter (b), and carotid flow rate (c) and pulsatility index (d) obtained by carotid ultrasonography with B‐mode (yellow arrow indicate carotid artery) and doppler ultrasonographic representative images (e). Protein content of eNOS (f) and VEGF (g) quantified as a percentage of HFD control group from the cortex region of the brain and representative western blots (h). Data expressed as mean ± SD. Data were assessed for normality using the Shapiro–Wilk test and visualized with a normal QQ plot. Time‐course measurements were compared by two‐way ANOVA with Tukey post hoc test where appropriate (b–d) while all protein measurements were compared by one‐way ANOVA with Tukey post hoc test where appropriate (e, f). Although one dataset did not meet normality according to the Shapiro–Wilk test (c), visual inspection of the QQ plot indicated only a single outlier, and the data were therefore treated as approximately normally distributed. Bolded *p* values are indicated for comparisons that reached statistical significance (*p* < 0.05). eNOS, endothelial nitric oxide synthase; HFD, high fat diet; NIT, nitrate; RN; RSV + NIT; RSV, resveratrol; VEGF, vascular endothelial growth factor; VTI, velocity time interval.

Given that 8 weeks of HFD feeding promoted the development of glucose intolerance and reduced carotid blood flow, we next aimed to investigate if +NIT and + RSV supplementation could rescue these impairments. To gain a comprehensive understanding of the temporal response and elucidate the progressive effects of our dietary interventions on blood flow, we conducted assessments at multiple time points throughout the study (2‐, 4‐, 6‐, and 8‐weeks; illustrated in Figure 3a). Analysis of carotid artery diameter revealed progressive changes in HFD‐fed animals over time. Post‐hoc analysis demonstrated significant decreases at each time point compared to 2 weeks (Figure 3b top, *p* < 0.05), with an additional decrease between 4 weeks and later time points (Figure 3b top, *p* < 0.05). At these later time points, HFD‐fed animals displayed significantly reduced vessel diameter compared to those supplemented with +NIT (Figure 3b bottom, *p* = 0.033) and showed a trend towards reduction compared to +RN (Figure 3b bottom, *p* = 0.095). Notably, the left carotid flow rate demonstrated decreased flow in HFD animals following 8 weeks (Figure 3d top), a time point where +NIT independently and combined with +RSV (+RN) prevented the ~20% HFD‐induced reduction in blood flow (Figure 3c bottom, *p* = 0.021 and *p* = 0.042, respectively). In addition to flow rate changes, we evaluated carotid artery pulsatility as a measure of vascular health. Consistent with the reductions in flow rate, the pulsatility index of the HFD group significantly decreased after 8 weeks (Figure 3d top, *p* = 0.009 vs. 2 weeks). These animals exhibited lower pulsatility compared to those supplemented with +RSV or + NIT, either independently or in combination (Figure 3d bottom, *p* = 0.026, *p* = 0.007, *p* = 0.004 respectively). Although the pulsatility index in the +RN group decreased at 6 weeks, it returned to baseline at the 8‐week time point. To examine whether dietary +NIT and +RSV influence vascular signaling pathways, we measured markers of angiogenesis in the cortex at the 8‐week time point. While eNOS protein remained unchanged across groups (Figure 3f, *p* > 0.10), analysis of VEGF protein content revealed significant treatment effects. Mice supplemented with +NIT showed significantly higher VEGF protein levels compared to unsupplemented HFD (Figure 3g, *p* < 0.05) while +RSV supplementation resulted in elevated VEGF protein content that approached statistical significance (Figure 3g, *p* = 0.062). Together with the previously observed HFD‐mediated reduction in carotid blood flow and cortex VEGF protein content (Figure 1), these data highlight the potential for dietary +NIT to preserve blood flow to the brain when challenged with a HFD.

### 3.4 The effects of nutritional supplementation with +NIT and +RSV on mitochondrial bioenergetics and markers of metabolic homeostasis

**FIGURE 4:**
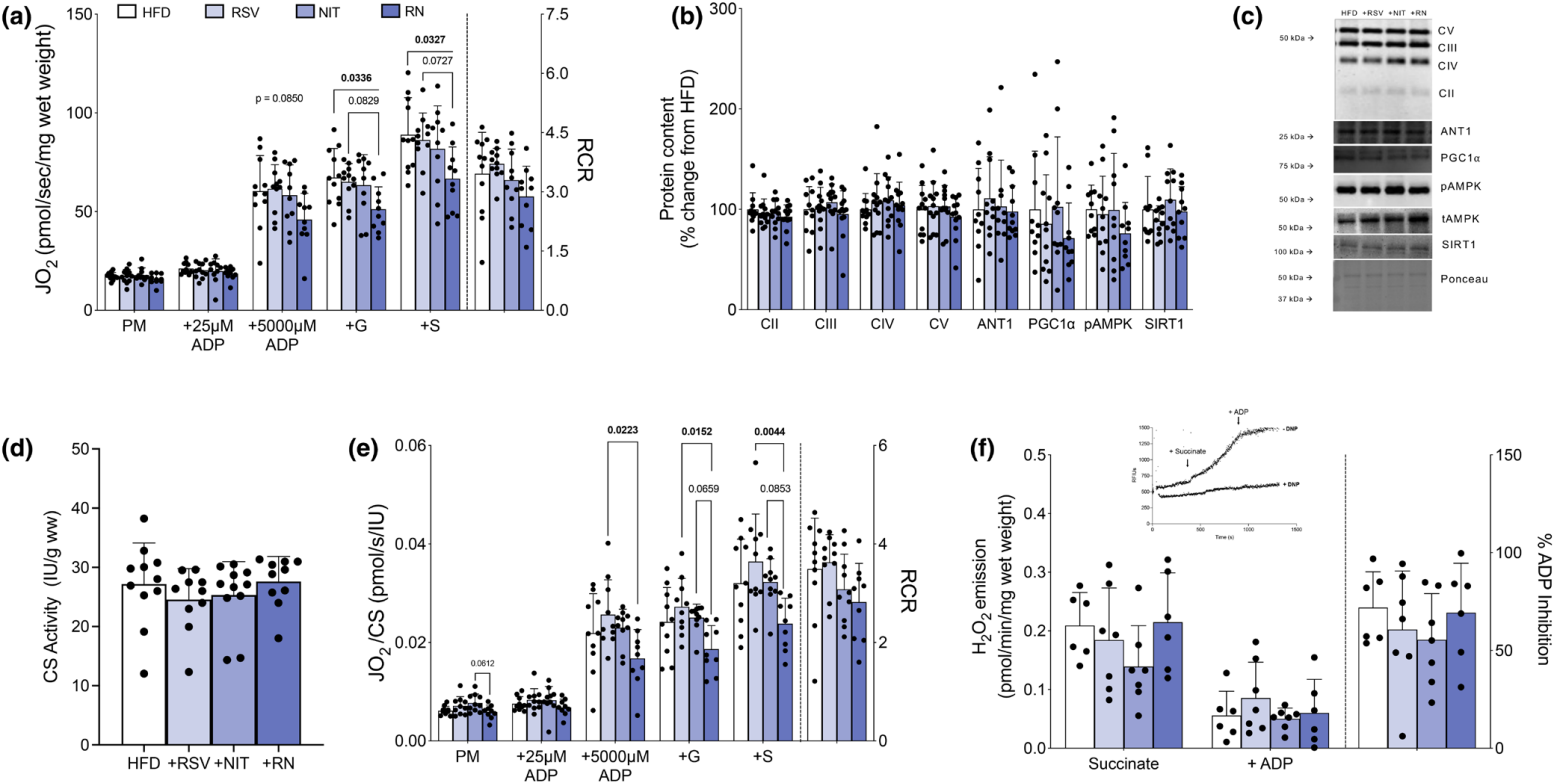
Supplementation with + RN reduces maximal complex I and complex II linked respiration in the cortex of the brain (*n* = 6–12/group). Mitochondrial respiration in the presence of various submaximal ADP and saturating complex I‐ and II‐linked substrates normalized to tissue wet weight (a). Protein content of OXPHOS complexes (CII, CII, CIV, CV), ANT1, PGC1α, the ratio of phosphorylated: total AMPK, and SIRT1 (b) quantified as a percentage of HFD control group (b) and representative western blots (c). Citrate synthase (CS) activity (d) and mitochondrial respiration in the presence of various submaximal ADP and saturating complex I‐ and II‐linked substrates normalized to CS activity (e). Measurements of mitochondrial H_2_O_2_ production in the presence of succinate and attenuation with the addition of ADP (f). Inset of a representative trace (*n* = 1/condition) in the presence or absence of mitochondrial uncoupler, DNP (f). Data expressed as mean ± SD. Data were assessed for normality using the Shapiro–Wilk test. Data that passed the Shapiro–Wilk test were analyzed using one‐way ANOVA followed by Tukey post hoc test where appropriate while data that did not pass the Shapiro–Wilk test were analyzed using Kruskal‐Wallis test followed by Dunn's multiple comparison test (B – CII, CIII, CIV, ANT1, PGC1α; D; E – +G). Bolded *p* values are indicated for comparisons that reached statistical significance (*p* < 0.05). +G, + glutamate; +S, +Succinate; ADP; adenosine diphosphate; AMPK, 5′‐adenosine monophosphate‐activated protein kinase; ANT1, adenine nucleotide translocase 1; HFD, high fat diet; JO_2_, rate of oxygen consumption; NIT, nitrate; OXPHOS, oxidative phosphorylation; PGC1α, peroxisome proliferator‐activated receptor gamma coactivator 1‐alpha; PM, pyruvate + Malate; RCR, respiratory control ratio; RN, RSV + NIT; RSV, resveratrol.

**FIGURE 5:**
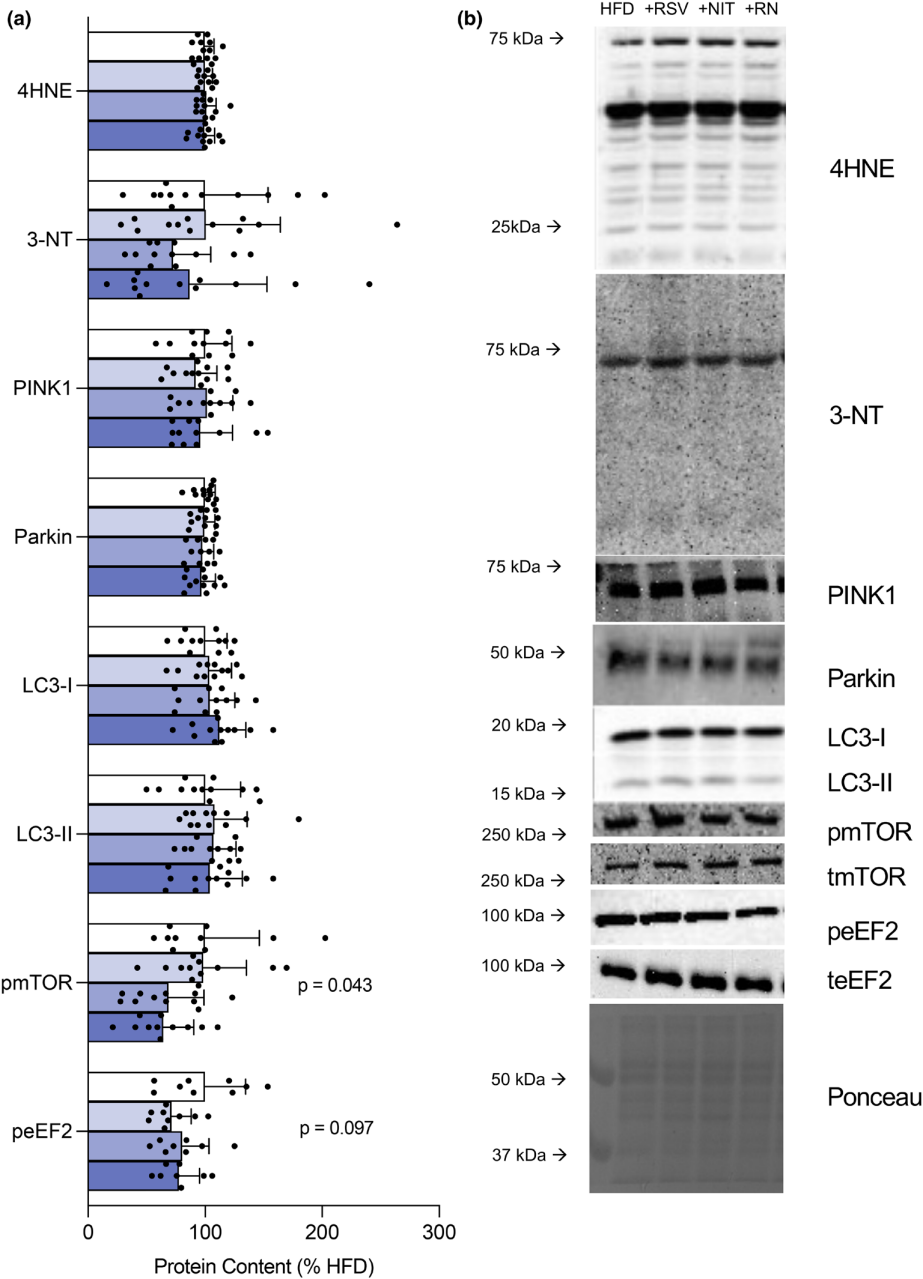
Supplementation with + RN does not alter markers of redox stress nor protein homeostasis in the cortex of the brain (*n* = 8–12/group). Protein content of 4HNE‐modified protein, 3‐NT, PINK1, parkin, LC3‐1, LC3‐II, as well as the ratio of phosphorylated: Total mTOR and eEF2 (a) quantified as a percentage of HFD control group in the cortex of the brain and representative western blots (b). Data expressed as mean ± SD. Data was assessed for normality using the Shapiro–Wilk test. Data that passed the Shapiro–Wilk test were analyzed using one‐way ANOVA followed by Tukey post hoc test where appropriate while data that did not pass the Shapiro–Wilk test were analyzed using Kruskal‐Wallis test followed by Dunn's multiple comparison test (A – 3‐NT, PINK1, LC3‐II, pmTOR). eEF2, eukaryotic translation, elongation factor 2; HFD, high fat diet; LC3, microtubule‐associated protein 1A/1B light chain 3; mTOR, the mammalian target of rapamycin; NIT, nitrate; 3‐NT, nitrotyrosine; PINK1, PTEN‐induced kinase 1; PGC1α, peroxisome proliferator‐activated receptor gamma coactivator 1‐alpha; RN, RSV + NIT; RSV, resveratrol; SIRT1, sirtulin 1; 4HNE, 4‐hydroxynonenal.

To understand how these bioactive nutritional compounds might influence brain energy metabolism, considering the observed changes in blood delivery via the carotid artery following 8 weeks of intervention, we comprehensively assessed mitochondrial function and related signaling pathways in cortical tissue. In contrast to our hypothesis, supplementation with +RSV and + NIT individually did not change mitochondrial respiratory capacity, while combined +RN reduced maximal Complex I‐ and II linked respiration compared to unsupplemented HFD (Figure 4a, *p* < 0.05) and trended lower compared to +RSV alone (Figure 4a, *p* = 0.083 and *p* = 0.073 for CI and CI + CII, respectively). Further, mitochondrial proteins (OXPHOS and ANT1), key regulators of mitochondrial biogenesis (PGC1α, pAMPK: tAMPK and SIRT1) and CS activity were not different between groups (Figure 4b–d, *p* > 0.10). Additionally, mitochondrial respiration normalized to CS activity remained lower following the combined +RN treatment compared to +RSV alone (Figure 4e, *p* < 0.05) and trending compared to +NIT alone (Figure 4e, *p* = 0.066 and *p* = 0.085 for CI and CI + II respectively) suggesting an intrinsic change within mitochondrion. Notably, these changes in respiratory capacity did not translate to differences in mitochondrial H_2_O_2_ emission or the ability of ADP to suppress H_2_O_2_ production (Figure 4f, *p* > 0.10), indicating maintained redox regulation despite reduced respiratory capacity. In support of unaltered redox balance following supplementation, we found no differences in markers of oxidative stress (4HNE and 3‐NT) between groups (Figure 5a, *p* > 0.10). Additionally, protein content of PINK1, Parkin, LC3‐I and LC3‐II remained unchanged between groups (Figure 5a, *p* > 0.10). While statistical analysis revealed an interaction effect for the ratio of phosphorylated: total mTOR (*p* = 0.043), post hoc comparison revealed no significant differences between groups. Similarly, the ratio of phosphorylated: total eEF2 showed trending, but not statistically significant differences between groups (*p* = 0.097). Collectively, these results suggest that combined supplementation with dietary NIT and RSV in the presence of HFD does not markedly affect markers of mitophagy, autophagy, or protein synthesis signaling within the cortex.

### 3.5 The effects of +NIT and +RSV supplementation on behavioral outcomes

**FIGURE 6:**
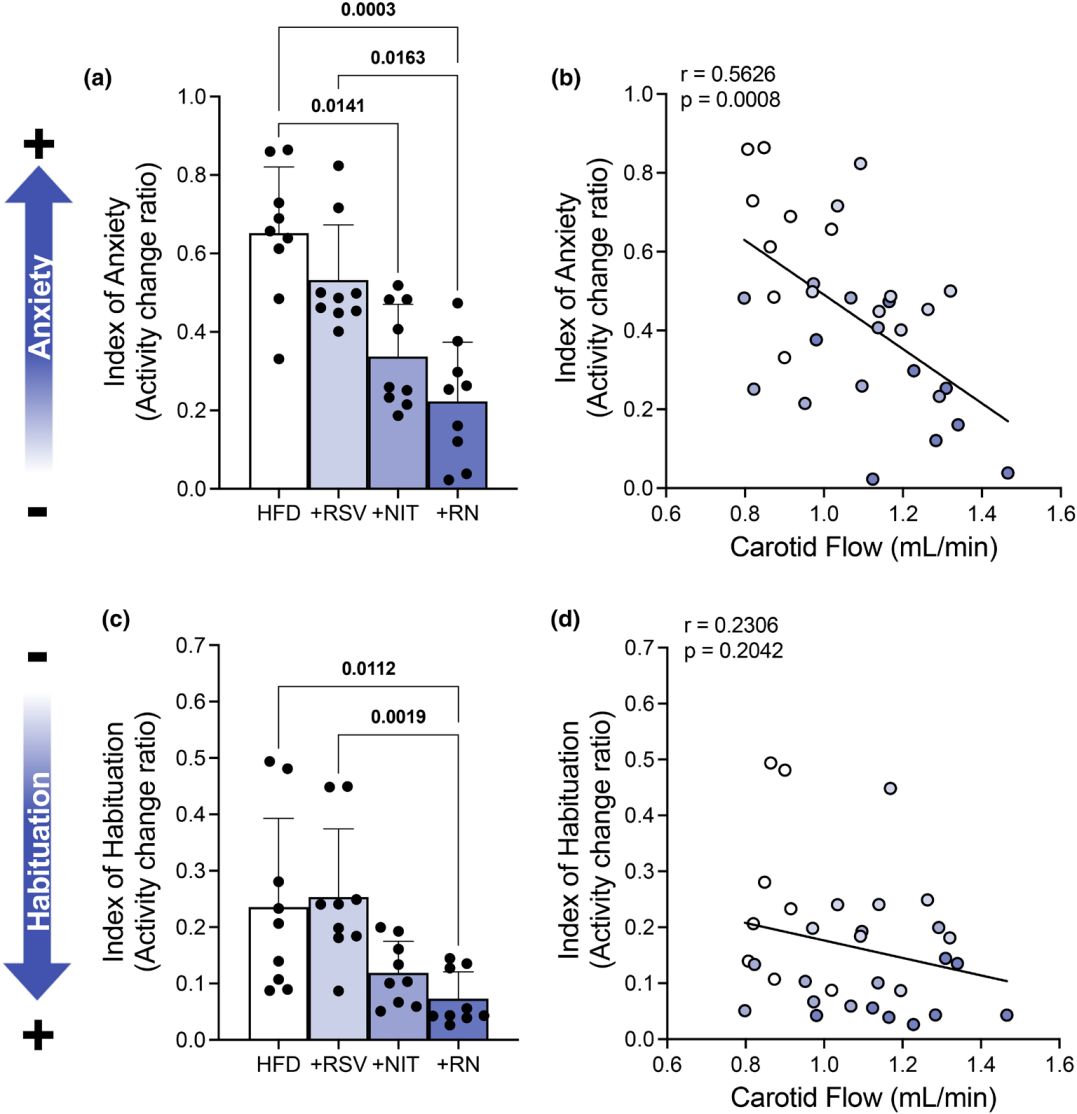
Supplementation with +NIT and +RN reduced anxiety‐like behavior and promoted greater habituation (*n* = 8–9/group). Activity change ratio for the total horizontal movement during the first 40 min (b) as an indicator of anxiety and a simple linear regression analysis was used to determine its association (*r*) with carotid blood flow. Activity change ratio for the total horizontal movement during the first 60–100 min (d) as an indicator of cognition and a simple linear regression analysis was used to determine its association (*r*) with carotid blood flow. Data expressed as mean ± SD. Normality testing using the Shapiro–Wilk test indicated that none of the data were normally distributed; therefore, group differences were evaluated using the Kruskal‐Wallis test followed by Dunn's multiple comparison test. Bolded *p* values are indicated for comparisons that reached statistical significance (*p* < 0.05). HFD, high fat diet; NIT, nitrate; RN, RSV + NIT; RSV, resveratrol.

Considering the apparent opposing changes in blood flow (increased) and mitochondrial OXPHOS (reduced) following combined +RN, we next assessed behavioral outcomes, specifically anxiety‐like behavior and habituation patterns, as functional readouts of our dietary intervention. Analysis of physical activity patterns during initial exposure to the novel environment revealed that both +NIT supplementation alone and in combination with +RSV (+RN) significantly reduced anxiety‐like behavior compared to HFD controls. These groups showed lower activity change ratios (Figure 6a, *p* < 0.05). Carotid blood flow in this subset of mice was also measured following 8‐weeks of dietary intervention and revealed significant inverse correlations between carotid flow and anxiety (Figure 6b, *r* = 0.563, and *p* = 0.001), indicating that increased carotid flow was associated with reduced anxiety‐like behavior. Assessment of habituation patterns revealed treatment‐specific effects. Analysis of total horizontal movement during the 60–100 min period demonstrated that while +NIT was not different than HFD‐fed animals (Figure 6c, *p* = 0.161), +RN treatment significantly improved habituation compared to both HFD and + RSV groups (Figure 6c, *p* = 0.011 and *p* = 0.002, respectively). However, no significant correlation was observed between carotid flow and habituation indices (Figure 6d, *r* = 0.231, *p* = 0.204). Together, these findings indicate that co‐supplementation of resveratrol and nitrate, produces beneficial effects on both anxiety‐like behavior and habituation in HFD‐fed mice, despite the observed reduction in mitochondrial OXPHOS, with anxiety improvements associated with increased carotid blood flow.

## 4 DISCUSSION

In the present study we provide evidence that dietary +NIT supplementation, alone or in combination with +RSV (+RN), prevents HFD‐mediated reductions in carotid blood flow, and improves anxiety‐like behaviors. These beneficial responses in the combined group (+RN) occurred despite a reduction in mitochondrial respiratory capacity within the cortex suggests a key role for blood flow to the brain in maintaining neurological health when metabolically challenged with diet‐induced obesity, and the beneficial effects of +RN supplementation.

### 4.1 +RN supplementation preserves carotid blood flow

Alteration in brain blood flow is increasingly recognized as a crucial factor in brain health during metabolic challenge. Our temporal analysis revealed that nitrate supplementation, alone or combined with resveratrol, preserved carotid blood flow, where +NIT and +RN maintained flow compared to the ~20% reduction observed in HFD‐fed mice. This preservation in blood flow was further reflected in our pulsatility index measurements where unlike HFD animals, all supplemented animals maintained pulsatility values following 8‐week intervention. The concurrent reduction in both parameters suggests that diminished blood flow rather than increased vascular resistance, drove the pulsatility changes in HFD mice. This vascular protection was likely associated with preserved VEGF protein content in both +NIT and + RN supplemented groups, suggesting maintained angiogenic signaling capacity. The parallel preservation of both VEGF content and carotid blood flow by +NIT supplementation suggests a potential relationship between these parameters and may provide insight into how nitrate maintains blood flow towards the brain via the carotid artery in HFD‐fed mice. While our measurement of carotid flow, pulsatility index, and VEGF protein content provide valuable insight into vascular function, direct assessment of cerebral perfusion and neurovascular coupling would be needed to fully characterize the effects of +RN on brain blood delivery. Nevertheless, these findings align with human studies demonstrating nitrate‐mediated increases in cerebral blood flow, particularly in the prefrontal cortex (Presley et al., 2011; Wightman et al., 2015), supporting the potential relevance of our vascular measurements.

### 4.2 Mitochondrial bioenergetics in the context of preserved blood flow

Alterations in mitochondrial bioenergetics within the brain is an emerging risk factor for cognitive impairments and accelerated age‐related cognitive decline. Indeed, genetic, and pharmacological models that increase mitochondrial content, mitochondrial respiration and/or reduce mitochondrial ROS have all provided compelling evidence for a key role of mitochondrial biology in maintaining neurological health (Angelova & Abramov, 2018; Calabrese et al., 2001; Hara et al., 2014; Norat et al., 2020; Rosenberg et al., 2023). As a major contributor to energy (i.e., ATP) and redox balance, alterations in mitochondrial bioenergetics are therefore thought to represent a key pathological event that precedes the development of numerous neurodegenerative diseases. However, in the present study HFD‐feeding did not alter indices of mitochondrial bioenergetics (i.e., respiration, OXPHOS capacity or markers of mitochondrial content), despite reducing carotid blood flow. While this highlights brain blood flow as the primary parameter affected by HFD, reduced perfusion likely created hypoxic conditions that influenced mitochondrial function in vivo, despite preserved capacity under optimal oxygen condition in vitro (Stepanova et al., 2019). Collectively, these data suggest that carotid blood flow changes may represent an early vascular response in diet‐induced obesity that precedes detectable intrinsic mitochondrial dysfunction.

Mechanistically, both +NIT and +RSV supplementation have been shown to elicit mitochondrial biogenesis properties in peripheral tissues via SIRT1‐AMPK‐PGC1 cellular cascade (Ashmore et al., 2015; Beaudoin et al., 2013; Lagouge et al., 2006), and therefore, we hypothesized that +NIT and +RSV supplementation would increase mitochondrial bioenergetics within the brain. However, in contrast to our hypothesis, +NIT and + RSV did not individually alter indexes of mitochondrial bioenergetics, including respiration, OXPHOS capacity, markers of mitochondrial content/biogenesis or mitochondrial H_2_O_2_ emission. It should be noted that our use of permeabilized intact tissues, while preserving native cellular architecture, may have obscured localized cellular responses or redox changes that would be apparent in isolated cell population. The consistent mitochondrial bioenergetics observed following independent supplementation may be attributed to the limited capacity for cellular renewal in adult cortex, as neural turnover in the brain is lower compared to peripheral tissues (St. John et al., 2016; Nesti et al., 2009). However, combined +RN manifested with decreased mitochondrial respiration within the cortex despite unchanged mitochondrial content and CS activity which suggest these results are due to intrinsic mitochondrial alterations. This observation may reflect the complex interactions between these compounds, particularly as nitrate‐enhanced blood flow could increase RSV delivery to neural tissue. While the mechanism‐of‐action remains unknown, previous research has shown that RSV can inhibit mitochondrial respiration within the brain at complex I and III, particularly at higher dosages (Gueguen et al., 2015; Moreira et al., 2013; Rowlands et al., 2015; Zini et al., 1999), suggesting that improved bioavailability through enhanced perfusion with nitrate might amplify these effects. Alternatively, we have previously shown that co‐supplementation (+RN) promotes lipid redistribution from white adipose tissue to peripheral tissues, a response that could potentially challenge the BBB integrity and manifest in stress‐pathway processes that reduce OXPHOS (de Paula et al., 2021; Handy et al., 2023; Zhang et al., 2013). While we have not directly measured BBB integrity in the present study, examining this parameter in our diet‐induced obesity model and the potential protective effects of supplementation represents an important future direction that could provide valuable mechanistic insights. Importantly, the reduction in respiratory capacity occurred without compromising redox balance or protein homeostasis, as evidenced by maintained H_2_O_2_ emission, markers of oxidative stress and markers of protein homeostasis.

### 4.3 Blood flow, and behavioral indices: Functional outcomes of +RN supplementation

The anxiety‐like behavioral improvements observed with +NIT and +RN supplementation are correlated with the preservation in carotid blood flow. Analysis of physical activity/exploration during novel environment exposure revealed that both +NIT and +RN significantly reduced anxiety‐like behavior compared to HFD controls, with these improvements strongly correlated with increased carotid blood flow. Additionally, +RN treatment significantly improved habituation patterns, although these improvements showed no direct correlation with blood flow measurements. It should be noted that behavioral analysis was not conducted on LFD animals due to confounding weight differences between groups and overall differences in activity throughout the CLAMS assessment (~24 h). Our analysis was therefore limited to within‐HFD comparison, allowing us to evaluate supplementation effects only relative to unsupplemented controls and cannot assess whether our dietary interventions restored normal behavioral function. However, the literature supports an association between high saturated fat intake and anxiety like‐behavior in mice (Fulton et al., 2022). Additionally, while our habituation assessments primarily reflect hippocampal‐dependent responses, the improvements observed likely extend to cortical function given the interconnectivity between these brain regions and the enhanced blood flow and preserved VEGF content we observed in cortical tissue likely indicate broader improvements in brain function. Nevertheless, an abundance of literature has demonstrated a clear link between OXPHOS on neurological health (Schimanski & Barnes, 2010; Tapella et al., 2022), and therefore the long‐term implications of the observed reductions in mitochondrial OXPHOS following combined supplementation remain unknown.

## 5 CONCLUSIONS AND FUTURE DIRECTIONS

Overall, the present data established the ability of +NIT, alone and in combination with +RSV, to maintain brain blood flow, markers of angiogenesis and habituation in the presence of an HFD. The protective effects of +RN were achieved without influencing mitochondrial ROS emission, redox stress, or markers of autophagy in the cortex. The strong correlation between preserved carotid blood flow and reduced anxiety‐like behavior despite reduced mitochondrial function, emphasizes cerebral perfusion as a potentially more immediate determinant of brain health during metabolic challenge than intrinsic mitochondrial capacity. Indeed, the present data highlights the need to interrogate mitochondrial bioenergetics and brain blood flow in an integrative manner in diverse situations, including, but not limited to stress and neurodegenerative diseases. Understanding the coordinated physiological outcomes, and mechanisms involved will likely contribute significantly to advancing our knowledge and therapeutic strategies for maintaining brain health.

## AUTHOR CONTRIBUTIONS

The overall conceptualization of studies included in this work was done by Geneviève J. DesOrmeaux, Rachel M. Handy, and Graham P. Holloway. All authors organized, performed experiments, analyzed, and interpreted the data; Geneviève J. DesOrmeaux, Jessica L. Dowling, and Graham P. Holloway drafted the manuscript. All authors approved the final version.

## FUNDING INFORMATION

This work was funded by a Natural Sciences and Engineering Research Council of Canada (NSERC) grant to Graham P. Holloway. Geneviève J. DesOrmeaux and Pierre‐Andre Barbeau were funded by an NSERC Doctoral Scholarship (CGS‐D). Rachel M. Handy and Jessica L. Dowling were funded by an Ontario Graduate Scholarship (OGS).

## CONFLICT OF INTEREST STATEMENT

The authors declare no conflicts of interest.

## Data Availability

Data will be made available upon reasonable request.
